# Alcohol use disorder as a risk factor for violent offending in a sample of female forensic-psychiatric inpatients

**DOI:** 10.1192/j.eurpsy.2022.883

**Published:** 2022-09-01

**Authors:** I. Franke, J. Streb, M. Dudeck, J. Mayer, I. Steiner, V. Wolf

**Affiliations:** 1 Ulm University, Forensic Psychiatry And Psychotherapy, Guenzburg, Germany; 2 kbo-Isar-Amper-Clinics Taufkirchen (Vils), Department Of Forensic Psychiatry And Psychotherapy, Taufkirchen (Vils), Germany

**Keywords:** female offending, violence, Treatment, alcohol

## Abstract

**Introduction:**

Female gender is associated with a lower risk for aggressive behaviour and violent offending. Well established risk factors for aggressive behaviour are alcohol and other substance use, but previous studies focused mainly on male offenders and the general population. However, for therapeutic and prognostic reasons it is important to understand pathways to female offending.

**Objectives:**

To examine a sample of female forensic-psychiatric inpatients regarding the association of alcohol (AUD) and other substance use disorders (SUD) with violent offending (homicide, assault, robbery).

**Methods:**

We conducted a retrospective cohort study of 334 female patients discharged before 01.01.2019 from a secure psychiatric hospital in Germany.

**Results:**

In total, 72% of the patients with AUD committed a violent crime, leading to admission to secure psychiatric treatment. In comparison a statistically significant lower rate (19%) of the SUD group was convicted of violent offending. Over 70% of the participants with AUD had a family history of AUD, and over 83% experienced physical violence in adulthood. We found no group differences (AUD vs. SUD) regarding aggressive behaviour during inpatient treatment.

**Conclusions:**

According to our results, AUD compared to other SUD, is a significant risk factor for violent offending in women. A family background with AUD and a history of physical abuse might function as a risk factor for both: developing an AUD and violent offending. The comparable rates of aggression in both groups during inpatient treatment suggest that abstinence is a protective factor.

**Disclosure:**

No significant relationships.

